# Polylactic Acid Cellulose Nanocomposite Films Comprised of Wood and Tunicate CNCs Modified with Tannic Acid and Octadecylamine

**DOI:** 10.3390/polym13213661

**Published:** 2021-10-24

**Authors:** Matthew J. Dunlop, Ronald Sabo, Rabin Bissessur, Bishnu Acharya

**Affiliations:** 1 Faculty of Sustainable Design Engineering, University of Prince Edward Island, Charlottetown, PE C1A 4P3, Canada; mdunlop@upei.ca; 2 Department of Chemistry, University of Prince Edward Island, Charlottetown, PE C1A 4P3, Canada; rabissessur@upei.ca; 3 USDA Forest Service, Forest Product Laboratory, Madison, WI 53726, USA; ronald.sabo@usda.gov; 4 Department of Chemical and Biological Engineering, College of Engineering, University of Saskatchewan, Saskatoon, SK S7N 5A9, Canada

**Keywords:** cellulose nanocrystals, nanocomposites, tunicates, extrusion, polymer–matrix composites

## Abstract

Herein, a one-pot strategy was used to prepare hydrophobic cellulose nanocrystals (CNCs) surface-modified with tannic acid and octadecylamine. By this strategy, CNCs derived from wood (W-CNC) and tunicates (T-CNC) were modified in situ and incorporated into a polylactic acid (PLA) matrix using two methods, without first drying the CNCs. Films of PLA-CNC nanocomposites were prepared both by solution casting and by wet compounding in a thermo-kinetic mixer, followed by melt extrusion. Various properties of these PLA nanocomposites were evaluated herein, along with an assessment of how these properties vary with the type of CNC reinforcement. Cast films with a hybrid mixture of wood and tunicate CNCs displayed improved mechanical properties compared to either wood or tunicate CNCs, but extruded films did not show this hybrid effect. The water vapor permeability of the extruded nanocomposite films with 1% CNCs was reduced by as much as 60% compared to the PLA films. The composite films also showed enhanced biodegradation compared to neat PLA films. These results demonstrate that wet compounded PLA composites produced with wood or tunicate CNCs modified using a one-pot, water-based route have improved barrier and biodegradation properties, indicating a potential for packaging applications without having to dry the CNCs.

## 1. Introduction

In the pursuit of alternatives to non-renewable petroleum-sourced materials, growing interest has been directed toward renewable resources and their sustainable derivatives that possess similar performance characteristics. One of these resources is lactic acid, which, when polymerized to polylactic acid (PLA), produces a renewable plastic material that may serve as a sustainable alternative to non-renewable petrol sourced plastics [1,2]. The advantages of PLA include its natural biodegradability and versatility and that it is prepared from sustainable natural resources. Producing PLA uses approximately 65% less energy than producing conventionally used plastics, generates 68% fewer greenhouse gases, and, when incinerated, emits fewer toxic fumes than most petroleum-based counterparts [3]. While there are many advantages of PLA, there also exist disadvantages that must be addressed if PLA is to replace non-renewable plastics in everyday applications. One major disadvantage of PLA is its inferior performance as a barrier material. Another disadvantage is its subpar durability [4]. As the polymer is exposed to humidity, water molecules permeate the polymer, causing it to undergo hydrolysis reactions, which degrade its structure [5]. To address these issues, researchers are attempting to modify PLA with nanofillers that can reduce vapor transmission through the composite material, which may also slow hydrolysis-based degradation [6,7].

One of these filler materials is the well-known natural polymer cellulose, which is the most abundant natural polymer on Earth and is synthesized in several natural resources, including plants, algae, bacteria, and tunicates [8]. Cellulose has many sustainable derivatives, one of which consists of the nanoscale crystalline region of the cellulose polymer. These cellulose nanocrystals (CNCs) are rod-like in shape, have excellent mechanical properties and good thermal properties, and can be modified to disperse in both hydrophilic and hydrophobic polymer matrices. Previously, others have incorporated CNCs into the PLA matrix and have noted both mechanical improvements [9,10,11] and a reduction in vapor transmission [12,13,14,15]. To avoid the aggregation of the filler and to improve adhesion with the matrix, CNCs generally undergo a hydrophobic surface modification prior to dispersion in the hydrophobic PLA matrix [9,10,15,16]. Hydrophobic CNCs have been demonstrated to enhance the mechanical, thermal, and vapor barrier properties of PLA, properties which have historically represented pitfalls when PLA is considered as a replacement for conventional plastics [15,17]. Unfortunately, these hydrophobic CNC surface modifications often come with formidable economic and environmental shortcomings, limiting their industrial potential [18]. Natural, food-safe modification chemistries are also desirable.

In this work, we utilized environmentally friendly chemistry to modify CNCs with low-cost plant-derived tannic acid (TA) using a previously described method [19]. The modified TA-CNCs were then imparted with a hydrophobic coating derived from octadecylamine (ODA) [20] to enhance the compatibility of the resulting ODA–TA-CNCs with the hydrophobic PLA matrix [19]. To evaluate the effect of CNC morphology on composite properties, two CNC types were used—low aspect ratio (length/diameter ~13) CNCs derived from wood pulp (W-CNCs) and relatively high aspect ratio CNCs (length/diameter ~59) derived from tunicates (T-CNCs). Because previous research has indicated that binary mixtures of wood and tunicate CNCs may provide better performance than either type alone [21,22,23,24], nanocomposites with both W-CNCs and T-CNCs were also prepared. Monofiller CNC and hybrid multifiller CNC mixtures are then combined with PLA by solution mixing, casting, and evaporative drying to yield films whose mechanical properties were investigated. To better simulate industrial practices, we also prepared monofiller and hybrid multifiller composites of various compositions by utilizing a thermo-kinetic wet compounding technique, which evaporates water and compounds the composite in a single process. The resulting composites were then melt-mixed and extruded into thin films, after which their mechanical, thermal, morphological, and vapor transmission properties were assessed.

Since the one-pot hydrophobic CNC surface modification is water-based and the utilized TA and ODA are low cost, there is great potential to utilize these ODA–TA-CNCs in industrial applications, provided that the modified CNCs display adequate dispersion in hydrophobic melt-mixed composites. The ODA–TA-CNCs prepared herein have never been combined with PLA, and thus the properties of the resulting nanocomposite have not been documented. Additionally, to the best of our knowledge, there have been no reports in the literature of hybrid PLA–CNC composites that combine high aspect ratio T-CNCs and low aspect ratio W-CNCs in PLA-based composite materials. Therefore, the materials prepared in this work are novel in several ways, demonstrating the feasibility of a green, low-cost, one-pot, hydrophobic CNC surface modification, and providing insight into the unique properties afforded by the resulting PLA–CNC composites.

## 2. Materials and Methods

### 2.1. Materials

Wood- and tunicate-derived sulfuric acid-hydrolyzed CNCs were prepared by our previously established protocols [25]. Both the W-CNCs and T-CNCs display shear birefringence with no observed flocculation or aggregation. The PLA used in this work was provided by NatureWorks (Ingeo 4044D) and was approved by the United States Food and Drug Administration (FDA) for use in food packaging applications. It has previously been determined to have a number average molecular weight of 158 kg/mol, a weight average molecular weight of 310 kg/mol, a density of 1.24 g/cm^3^, and a melt flow rate of 0.395 g/min [26]. Additional materials, including ACS reagent-grade tannic acid (TA), octadecylamine (ODA) (97%), sodium hydroxide (NaOH) (98%), 200 proof ethanol, and 4-(2-hydroxyethyl)-1-piperazineethanesulfonic acid (HEPES) (99%), were purchased from Sigma Aldrich and used without modification.

### 2.2. CNC Modification

To impart hydrophobicity to the naturally hydrophilic CNCs and thus make them more compatible with the hydrophobic PLA, a water-based surface modification was utilized. As visible in Table 1, T-CNCs are over four times the aspect ratio of W-CNCs, resulting in higher viscosity dispersions for T-CNCs compared to W-CNCs at similar concentrations [27]. We accomplished our CNC modification utilizing a similar procedure to Zhen et al., who previously prepared TA-decylamine-modified W-CNCs at 1 wt.% [19]. However, given that T-CNCs are considerably more viscous than W-CNCs at this concentration [27], we chose to set the initial concentration of both W-CNCs and T-CNCs at 0.58 wt.%. This allowed us to maintain consistent mechanical stirring throughout the CNC modification. Outside of this change and the utilization of ODA (a C18 alkyl chain) rather than decylamine (a C10 alkyl chain), our modification was consistent with that of Zhen et al. [19] and is described further in Supplementary S1. We note that both W-CNCs and T-CNCs modified in this way precipitated after the addition of ODA, consistent with previous reports [19]. The original reaction suspension was used as an input for all subsequent composite preparations without any purification or post-treatment. This reaction is one pot, requires under 12 h, and utilizes mild reagents and simple mechanical agitation to produce hydrophobic CNCs.

### 2.3. Cast Film Preperation

To determine the compatibility of these ODA–TA-CNCs with PLA, we prepared solution-mixed cast films utilizing benzene as a common solvent. Vacuum-dried samples of PLA and ODA–TA-CNCs were dissolved in benzene at 60 °C and solution mixed to form tunicate, wood, and hybrid PLA–CNC composites at a 1 wt.% CNC loading. For comparison, a cast film PLA sample was prepared by dissolving PLA in benzene at 60 °C. Benzene was chosen as it fully dissolved the PLA sample and dispersed the modified CNCs under these conditions. All samples were dried in a fume hood at room temperature under identical conditions.

### 2.4. Extruded Film Preperation

#### 2.4.1. Wet Compounding

To prepare extruded films, suspensions of ODA–TA-modified CNCs were adjusted to either 1 wt.% or 2 wt.%, and then combined with unprocessed 2 mm PLA pellets in a thermo-kinetic mixer (k-mixer) in a 1:1 ratio. This resulted in PLA samples with 1 wt.% and 2 wt.% CNC loadings. Samples were initially mixed at 500 rpm for 30 s to ensure consistent mixing, after which time the mixer was ramped to 6000 rpm, in accordance with previously established protocols [28,29]. Frictional heat from the k-mixing process evaporated the water and melted the PLA. These concurrent processes within a high-speed mixing chamber lead to a sharp increase in the temperature and viscosity of the molten polymer mix immediately after evaporation of the water. This temperature ramp triggered the ending (140 °C cut-off) of our k-mixing process and the viscosity change served as an audible indicator preceding the automated k-mixing cut-off.

#### 2.4.2. Grinding

All k-mixed composites, a k-mixed PLA sample, and a pristine PLA sample were ground to powder prior to extrusion by passing liquid nitrogen-cooled samples through a Model 4 Wiley Knife Mill equipped with a 2 mm screen and operating at a constant rotation of 800 rpm. The samples were immersed in the liquid nitrogen for 10 min prior to grinding. These powders were collected and stored under vacuum until immediately prior to extrusion.

#### 2.4.3. Twin Screw Extrusion

Films were extruded using a DSM Xplore 15 mL microcompounder (DSM Research, Heerlen, The Netherlands). Vacuum-dried powdered samples (< 2 mm) were fed continuously into the compounder. The chamber was maintained at 180 °C and the screw speed was set to 50 rpm. The samples were then extruded through a heated rectangular dye maintained at 140 °C. Compressed air was used to cool the samples as they were collected using a Xplore Cast Film Device(Sittard, The Netherlands) operating at a speed setting of 500 mm/min and a torque setting of 50 Nmm. In this way, films were extruded and rolled into circular spools with film thicknesses between 0.2 and 0.4 mm, which were subsequently unspooled, cut into strips, and placed between metal plates to flatten prior to analysis.

### 2.5. Sample Designation

To differentiate the prepared samples, we designated sample codes as in Table 2.

The “1” and “2” in the sample code represent the loading of CNCs by wt.%; all CNCs used were assumed to be modified as described in Section 2.2. All hybrid mixtures were comprised of a 1:1 ratio of W-CNCs/T-CNCs. Two types of hybrid mixtures were prepared: (1) Combined before k-mixing (designated “Pre”) and (2) combined after k-mixing (designated “Post”). When the wood- and tunicate-derived CNCs were mixed prior to the k-mixing process (pre-k-mixing), we denote the resulting composite “Pre,” and when k-mixed samples of W-CNC- and T-CNC-reinforced PLA were combined post-k-mixing, in the grinding process prior to extrusion, we refer to these as “Post.” In this way, we were able to elucidate the differences between mixing the CNC types either pre- or post-k-mixing.

### 2.6. Statistical Analysis

The standard deviation was calculated for all data produced herein in accordance with the following equation:

(1) $$Standard\;Deviation=\sqrt{\frac{\sum_{i=1}^{n}{\left(x_{i}-\bar{x}\right)}^{2}}{n-1}}\;$$

where *n* is the number of data points in the data set, $\bar{x}$ is the mean value of the data set, and $x_{i}$ is the *i*^th^ point in the data set.

### 2.7. Experiment

#### 2.7.1. Contact Angle

Contact angle measurements were performed on a Biolin Scientific Attension Theta instrument(Alberta, Canada). Samples approximately 2 cm in length by 2 cm in width and 0.2 mm thick were mounted on the flat sample holder and oriented toward the high-resolution NAVITAR digital camera. After focusing the camera, a single drop of deionized water approximately 10 µL in volume was dropped onto the sample surface. The average contact angle on both sides of the sample, over a 5 min testing duration, was calculated using the instrument’s OneAttension Version 2.6 software. All tested samples were subjected to the same test method and were visually assessed for imperfections prior to analysis.

#### 2.7.2. FTIR

Attenuated total reflectance Fourier transform infrared Spectroscopy (ATR-FTIR) was performed on cast and extruded film samples approximately 0.2 mm in thickness using a Bruker Alpha FTIR spectrometer (Alpha-P) equipped(Milton, Canada) with OPUS Version 6.5 software. The measured transmittance values were determined using 32 averaged scans, with a 4 cm^−1^ resolution, corrected against a background scan to yield the reported spectra in the range of 4000–500 cm^−1^.

#### 2.7.3. TEM

The morphology of the modified T-CNC and W-CNC was assessed by obtaining transmission electron microscopy (TEM) micrographs on a JEOL 2011 STEM instrument(Saint-Hubert, Canada). Dilute (~0.001 wt.%) colloidal suspensions were cast onto etched copper-coated grids and air-dried prior to imaging. The average length, width, and aspect ratio were then calculated from at least 50 manual measurements from 5–10 representative micrographs of each sample using Image J Fiji software.

#### 2.7.4. SEM

The surface of the extruded films and the resulting surface erosion after biodegradation studies were studied by scanning electron microscopy (SEM). A DeLong LVEM5 instrument with an accelerating beam at a voltage of 5 kV was used. The magnification of all micrographs was 120× and the working distance was 5000 microns. Samples subject to biodegradation were first cleaned by soaking the sample in deionized water overnight, then scratching lightly with a smooth metal spatula and vigorously rinsing the samples under running DI water. The samples were then prepared by mounting dried film specimens on the brass sample holder using double-sided carbon tape. The specimens were then coated with a 60:40 gold/platinum alloy using a sputter coater prior to imaging.

#### 2.7.5. TGA

Thermogravimetric analysis (TGA) was utilized to determine the thermal decomposition profiles for all samples prepared by extrusion. Experiments were performed on a TA Instruments TGA Q500 under an oxidizing atmosphere (60 mL/min compressed air and 40 mL/min nitrogen) from room temperature to 600 °C, using a heating rate of 10 °C/min.

#### 2.7.6. DSC

Thermal properties were further assessed by utilizing differential scanning calorimetry (DSC). A DSC Q100 instrument was used under a 50 mL/min nitrogen purge. Samples weighing ~10 mg were crimped into aluminum pans and equilibrated in the DSC at 30 °C. Then, the samples were heated at 10 °C/min to a final temperature of 180 °C and cooled at 10 °C/min back to the initial temperature of 30 °C. In this manner, all samples were cycled 10 times from 30 to 180 °C and back to 30 °C.

#### 2.7.7. Tensile Testing

The mechanical properties of both cast and extruded film samples was determined by tensile testing of Type V dogbone-shaped specimens in accordance with ASTM D638, with minor modifications described here and in prior work [30]. We prepared 10 replicates of each extruded sample, but due to the limited size of the solution cast films, we prepared five replicates of each cast sample. All samples were punched from film samples using a Type V dogbone punch and a hydraulic press at approximately 1000 psi. Cast films were approximately 0.2 mm thick, and extruded films were approximately 0.4 mm thick. Samples were elongated at 1 mm/min utilizing an Instron Universal testing instrument at the Engineering Mechanics and Remote Sensing Laboratory (EMRSL) within FPL. A laser extensometer was utilized to track the sample extension more accurately. The average values and corresponding standard deviation values of the elastic modulus, maximum force, maximum strength, and toughness of the samples are reported. The elastic modulus was determined by EMRSL from the initial slope of a hyperbolic tangent curve fit to the stress–strain data. Toughness was determined by numerically integrating the area under the stress–strain curve.

#### 2.7.8. Vapor Transmission

Vapor transmission properties, including water vapor transmission (WVTR) rates, permeance, and permeability, were assessed in accordance with the ASTM E96 standard desiccant method B. Model 68-3000 E-Z Cups (Thwing-Albert, West Berlin, Germany) were utilized with 0.3 mm-thick film samples, which were sandwiched between metal gaskets with a 1 cm × 5 cm rectangular opening. Film specimens were conditioned for two weeks in a 70 °F and 50% RH controlled atmosphere room before being analyzed in the same environment. The thickness of the film samples was determined using a digital micrometer with 0.001 mm accuracy. The desiccant was calcium chloride (CaCl2) particles (1.0–2.0 mm) placed into an oven at 200 ℃ for 12 h and were then assumed to have uniform relative humidity (RH) of ~0%. Desiccant was added to the E-Z Cups to within 6 mm of the lower metal gasket, conditioned film specimens were mounted as described to the top of the E-Z Cups, vacuum grease was placed between the gaskets to ensure an airtight seal, and the lid was securely affixed. The entire assembly was agitated manually and weighed daily using a scale with a 0.001 g accuracy over a period of 30 days. Duplicate trials were performed on all samples and the resulting WVTR, permeance, and permeability values are an average of both trials with corresponding standard deviation. The equations used to calculate WVTR, permeance, and permeability are consistent with previously reported protocols [29,30,31].

#### 2.7.9. Biodegradation

Biodegradation of the extruded samples prepared in this work was performed in accordance with ISO 14853, with minor modifications described here. Ten replicates of each sample were prepared for analysis as square shapes with a length and width of 1 cm and a thickness of 0.4 mm. The laboratory-scale reactor designed for this study was a 10 L polyethylene vertical cylindrical container with a height of 12 inches and a diameter of 8 inches. The working volume was ~9 L. The contents of the reactors were mixed with a magnetic stirrer. The temperature was maintained at mesophilic temperature (35 °C) [32,33] with an insulated heating jacket controlled by a PID (proportional–integral–derivative) controller. The reactor was equipped with two ports—one which was used as the gas outlet and the other for feeding and content removal. The reactor was initially fed with inoculum (1 L) and sludge (3 L) from the Charlottetown Pollution Control Plant (CPCP) located in PEI, Canada. The system was then fed with a mixture of sludge, lactose, and starch at a ratio of 1:1:2 in 200 mL of water as substrate every other day. Once the working volume of 9 L was reached, 200 mL of the digestate was removed and an equal volume of fresh substrate was introduced. The whole system was operated for a period of 30 days. The gas production was monitored to ensure working of the reactor, using a syringe installed on the gas outlet line. The required nutrients and micronutrients necessary to facilitate and promote bacterial growth were assumed to be present in the sludge obtained from the CPCP.

## 3. Results and Discussion

### 3.1. Hydrophobic CNC Modification

**Figure 1:**
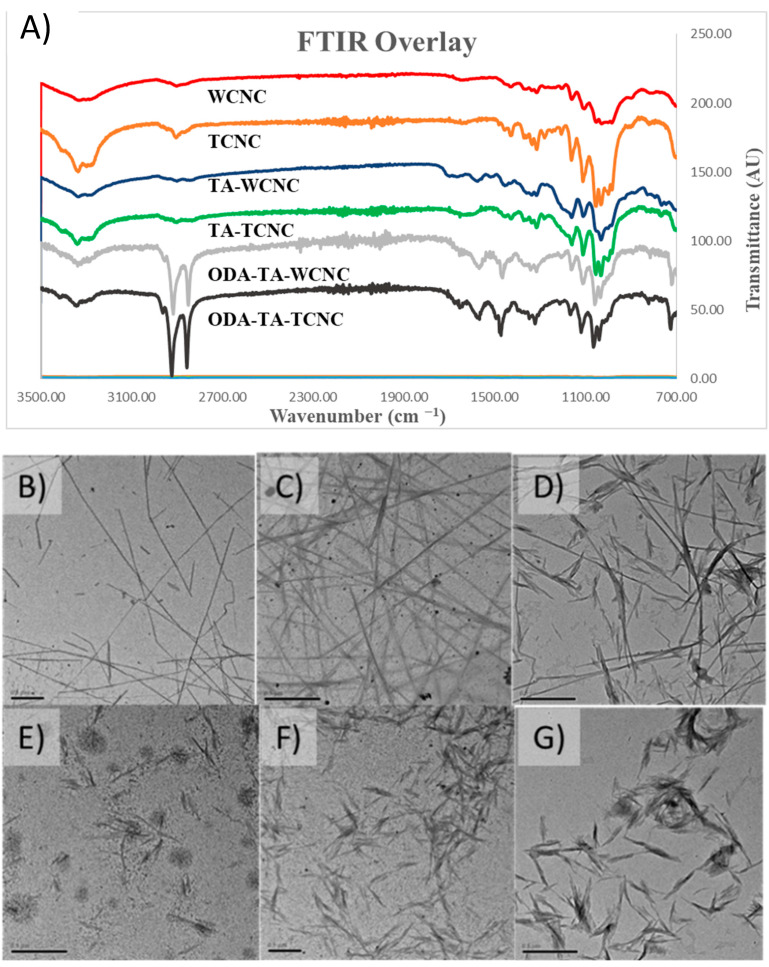
(**A**) Overlaid FTIR spectra of the prepared ODA–TA-CNCs and their precursors: (**B**,**E**) T-CNCs and W-CNCs, respectively; (**C**,**F**) TA-T-CNCs and TA-W-CNCs, respectively; (**D**,**G**) ODA–TA-T-CNCs and ODA–TA-W-CNCs, respectively. Note: The scale bar in each micrograph is 500 nm in length.

Successful modification of the T-CNCs and W-CNCs was determined by three complementary techniques described here and in Supplementary S2. The contact angle measurements confirmed that the hydrophobicity of the CNC samples increased from roughly 20° to over 70° after the ODA–TA modification (See Supplementary S2). The presence of both asymmetric (2860 cm^−1^) and symmetric (2940 cm^−1^) CH_2_ stretches from the C18 alkyl chain combined with both primary (1560 cm^−1^) and secondary (1490 cm^−1^) N–H bending signals present in the FTIR data indicates that this increased hydrophobicity of the modified CNCs was due to the presence of ODA on the surface of the TA-CNCs [19,34,35]. In the latter case, N–H bending is attributed to the ODA reacting with the TA-CNCs through a Michael-type addition. However, the ODA may also react with the TA-CNCs via a Schiff base formation, which yields a tertiary amine and would not possess an N–H signal. These are the same concurrent reaction mechanisms proposed by Zhen et al. for the formation of decylamine–TA-CNCs [19], and is consistent with the ODA modified polydopamine reported by Wang et al. [35]. Additionally, the TEM micrographs visible in Figure 1 and measured in Table 1 afford a direct means to measure the increasing width, which is expected by coating the CNC surface first with TA and then with ODA [36].

### 3.2. Thermal Analysis

#### 3.2.1. TGA and DTGA

**Figure 2:**
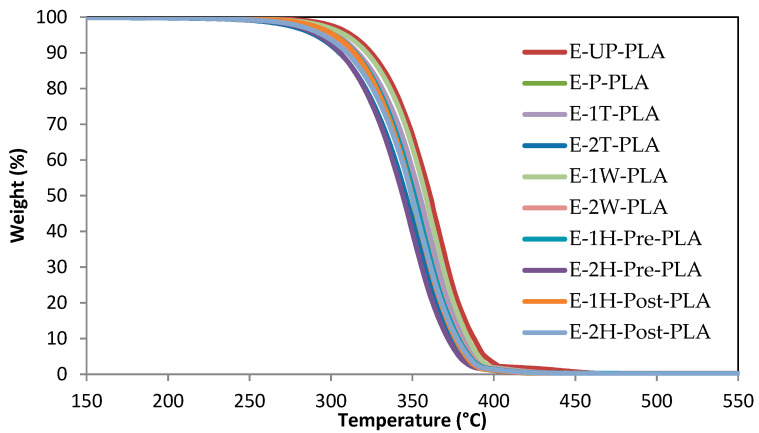
Thermal decomposition profiles of the designated composites.

The thermal properties of the extruded samples were assessed using TGA to determine their thermal decomposition profiles in an oxidizing atmosphere. All samples analyzed in this way displayed similar decomposition profiles (Figure 2) consisting of a single mass loss event between 250 and 400 °C [15,37,38]. No significant difference was observed between the monofiller and the hybrid multifiller composites, although it is notable that the samples containing a 2% CNC loading decomposed slightly earlier than those with a 1% CNC loading, as well as the PLA. This was also true of the rate of maximum mass loss, which is clearly seen in Supplementary S3 and is recorded in Table 3 as the inflection point of the DTGA thermograms.

#### 3.2.2. DSC

**Figure 3:**
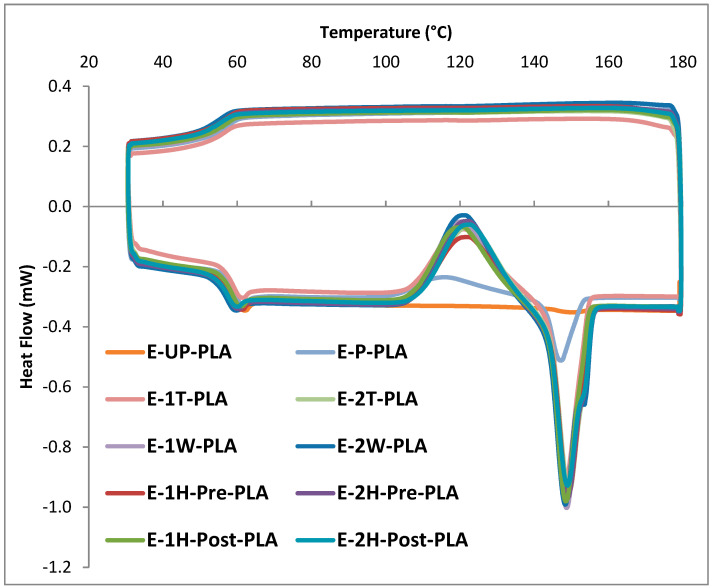
Overlaid DSC thermograms of composites from the second thermocycle.

The thermal properties of the nanocomposites were evaluated by DSC. The glass transition temperature (Tg), the crystallization temperature (Tc), the enthalpy of the crystallization (ΔHc), the melting temperature (Tm), and the enthalpy of the melt (ΔHm) were assessed for both the first thermocycle and for later thermocycles (which remained relatively consistent for the 2nd–10th cycles). The differences between samples, as well as the changes that the samples underwent between their first thermocycle and later cycles, were assessed. The results from the second cycle are shown in Figure 3 and Table 4. The results from the first cycle, including changes between the first and second cycles, are shown in Supplementary S4.

Notably, the first cycle of heating and cooling produced values that were considerably different from the second heating cycle in all samples. This is due to the polymeric samples conforming to their sample pans within the DSC in the first thermocycle. In all cases, the thermograms were consistent from the 2nd to the 10th cycles. The rate of change in the Tg between the first cycle and later cycles was observed to be greatest for composites with a 2% CNC loading. The Tc and ΔHc either increased in magnitude or were first observed in the second heating cycle, and remained consistent in later cycles. Additionally, the Tm decreased in temperature and the ΔHm decreased in magnitude from the first to the second cycle.

The PLA extruded films (E-UP-PLA) behaved markedly different than the other samples, without clear crystallization or melting temperatures. The k-mixed PLA control films (E-P-PLA) clearly displayed these properties, at lower temperatures than the PLA-CNC composites, however. Therefore, the k-mixing process likely either modified the PLA structure or introduced contamination, which resulted in crystallization that was not observed for films produced by extruding neat PLA resin, consistent with past reports [39].

We assessed that the presence of modified CNCs provided nucleation points, which resulted in significant crystallization, especially compared to unfilled PLA [40,41,42]. We also observed that the Tg was reduced for composites with a 2% CNC loading compared to composites with a 1% CNC loading in the PLA samples. Curiously, a second melting transition was observed at a slightly higher temperature in composites with a 2% CNC loading, and this was not seen in those composites with a 1% CNC loading. This is similar to the PLA–CNF composites reported by Frone et al. [43] and others [42], indicating a biphasic material. We posit that increased CNC aggregation leads to a heterogonous samples, where the thickness of the crystalline lamellae formed during cold crystallization may be sufficiently different between these two phases to yield separate melting transitions [44].

### 3.3. Mechanical Analysis

#### 3.3.1. Tensile Testing of Cast Films

**Figure 4:**
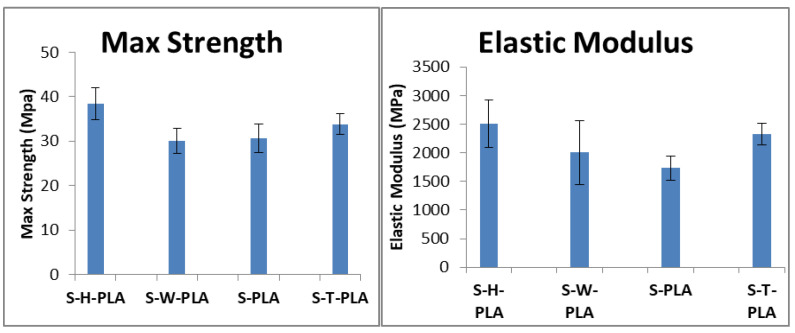
Measured mechanical properties of solution-cast film samples.

Although solvent casting necessitates solvent removal, which is costly, slow, and often incomplete, this technique allows for the organization of CNCs in the dispersion into ordered structured domains arising from particle–particle interactions between CNCs [18,25]. Tensile testing can provide some insight to this structure, as well as the interactions between CNCs and PLA. Figure 4 and Supplementary S5 show that in cast films, the hybrid CNC mixtures outperformed the monofiller CNC reinforcement in terms of strength, modulus, and toughness.

This is consistent with previous studies that demonstrated synergistic mechanical reinforcement in solution-cast polymeric composites reinforced with hybrid CNC mixtures, compared to monofiller CNC composites [23]. This “hybrid effect” was demonstrated previously with hydrophilic CNCs in a polar aqueous solution, and now in nonpolar benzene using hydrophobic CNCs. However, further investigation is warranted to determine the mechanisms responsible for the increased mechanical properties in these hybrid composites. It is not clear whether CNC–CNC interactions and organization are responsible for this effect in composites. Moreover, solution cast films are not scalable, and therefore, it is important to determine if any hybrid effect can extend to other more common composite preparation techniques such as melt mixing [18].

#### 3.3.2. Tensile Testing of Extruded Films

**Figure 5:**
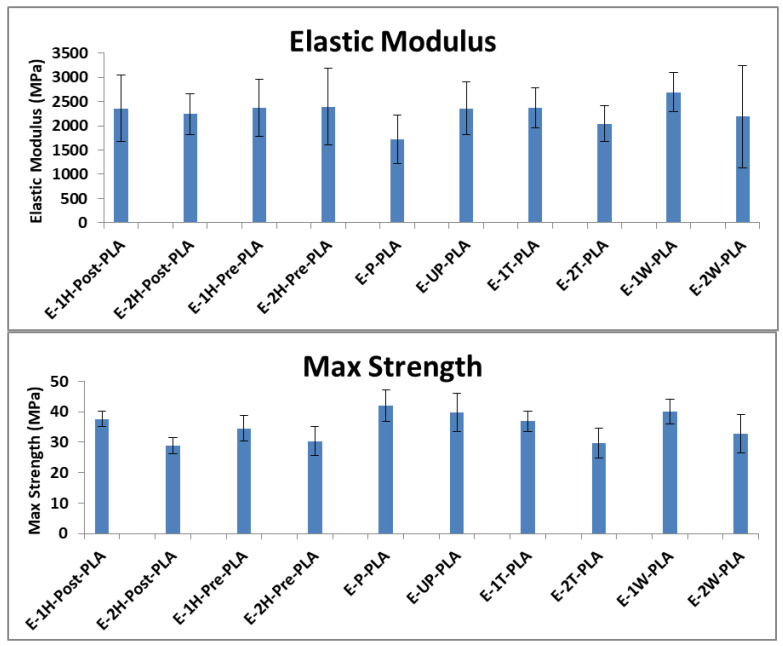
Measured elastic modulus and maximum strength of extruded film samples.

Composite samples were produced by k-mixing and extrusion to determine whether the unique mechanical properties exhibited by hybrid CNC mixtures in solution-cast samples would extend to samples prepared by melt processing. As documented in Figure 5 the strength, modulus, and maximum force values for all samples containing CNC were similar, and the hybrid mixtures did not show the same synergistic reinforcement observed in the solution cast samples. It is well known that melt-mixed polymeric composites, which incorporate nanofillers, are prone to suffering from aggregation of the nanofiller during the melt-mixing process [45]. We posit that the aggregation of CNC particles during melt mixing may have led to a reduced dispersion of the CNCs within the PLA matrix, and that this reduced dispersion degrades the mechanical reinforcement of the filler due to a lower filler–matrix contact area and the inability of the aggregated filler to percolate the PLA matrix [46,47]. This CNC aggregation prohibits hybrid CNC mixtures from providing the synergistic mechanical reinforcement observed in solution-cast films. Further evidence of this aggregation is provided by comparing the mechanical properties of the composites with different CNC loadings. The 2 wt.% CNC composites, which contained more CNC reinforcement, consistently underperformed the 1% CNC composites, which contained less reinforcement. Since stress transfer from the matrix to reinforcement is governed by their contact area, this suggests that CNC aggregation accelerates with increased CNC loadings, leading to reduced mechanical properties despite the increased CNC loading.

### 3.4. Vapor Transmission

**Figure 6:**
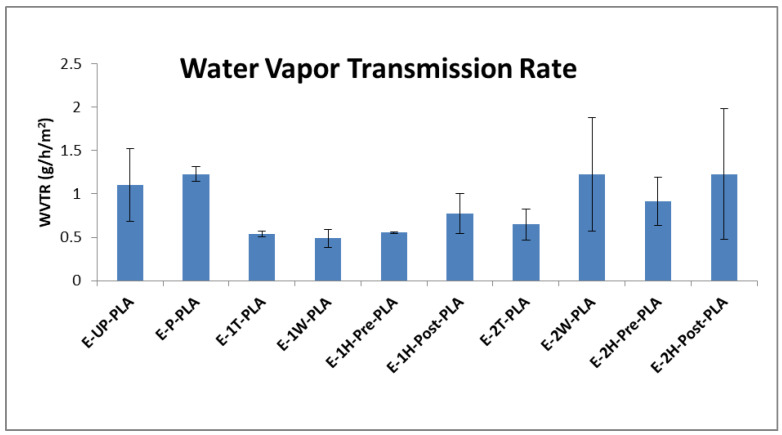
Measured water vapor transmission rates of all extruded film samples.

The NatureWorks Ingeo 4044D grade of PLA used in this work has already been approved by the United States Food and Drug Administration (FDA) for use in food packaging applications [48], and a variety of nanocellulose materials are also finding use in this area [49]. Presented here for the first time is the effect of the ODA–TA-modified CNCs on the vapor transmission properties of PLA films. The water vapor transmission rate (WVTR) data is visible in Figure 6, the permeance and the permeability data is visible in Table 5 and in Supplementary S6 of the Supplementary Information.

The addition of modified CNCs to the PLA matrix at 1 wt.% led to a reduction in vapor transmission rates, permeance, and permeability compared to both pristine and k-mixed PLA standards. For nanocomposite films containing 2 wt.% wood CNCs or 2 wt.% hybrid wood–tunicate CNCs, the barrier properties were more comparable to the PLA without CNC addition, which may relate to the increased aggregation of the CNC filler at a higher CNC content. This indicates that the 1 wt.% CNC composites may be more suitable for use in food packaging applications than composites with 2 wt.% CNC.

### 3.5. Biodegradation

**Figure 7:**
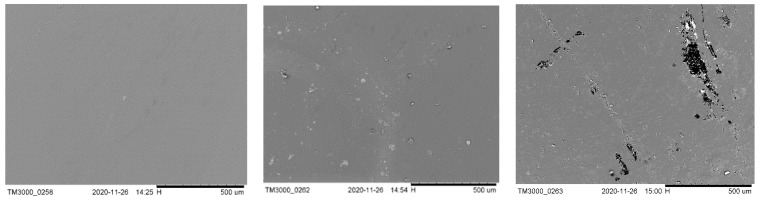
Pristine E-P-PLA sample (**Left**), E-P-PLA sample after anaerobic digestion (**Center**), and E-2T-PLA sample after anaerobic digestion with visible surface erosion (**Right**). The scale bar (black, bottom right) in all micrographs is 500 microns in length.

To understand how the biodegradation of the studied materials differs with changing CNC compositions and loadings, we preformed anaerobic digestion on all samples prepared via extrusion. The bioplastic samples were then analyzed for surface erosion utilizing SEM, followed by a gravimetric determination of weight loss. The resulting SEM micrographs and calculated weight loss are presented in Table 6 and Figure 7, respectively.

In the environment, PLA waste is primarily degraded by mesophilic bacteria [32], which have been known to exist in the wastewater sludge of pollution control plants [33]. By harvesting wastewater sludge and inoculum from a local pollution control plant, we successfully demonstrated that PLA composites reinforced with ODA–TA-CNCs can also be biodegraded by utilizing these inputs. As clearly visible in the SEM micrographs, anaerobic digestion led to visible surface erosion in PLA-based bioplastic samples. Interestingly, there seems to be a correlation between the presence of CNC in the composites and the resulting surface erosion, with all CNC-containing composites displaying enhanced biodegradation compared to the PLA samples without CNCs. This may be attributable to the mechanism described by Shanmuganathan et al., which posits that CNCs act as channels that allow solvent to more efficiently penetrate the composite compared to the matrix material without CNCs [46,50]. We first note that composites loaded with T-CNCs had more remaining mass than those with wood, although the variability was too high for statistical certainty. Tunicate CNCs have been reported to have a natural resistance to biodegradation [51], so further study is needed to evaluate the biodegradation of PLA-T-CNC composites. Similarly, the composites containing 2 wt.% CNC also appear slightly more susceptible to biodegradation, which may be related to the increased aggregation observed in these samples, which is visible in Supplementary S7. While the overall biodegradation of our samples was relatively low, with a maximum mass loss of ~10 wt.% after one month, our findings indicate that ODA–TA-CNCs can improve the biodegradability of PLA. The limits of this improvement may be understood through future work further exploring the biodegradation of these materials under both mesophilic and thermophilic conditions.

## 4. Conclusions

A one-pot, water-based strategy was used to modify both wood and tunicate cellulose nanocrystals with tannic acid and octadecylamine. PLA–CNC composites containing CNCs from wood, tunicate, or a combination of both were produced by using solvent casting and melt extrusion. Films that were produced by melt extrusion were first compounded in the presence of water in a thermo-kinetic mixer, which both evaporated the water and compounded the PLA and CNCs. The modified CNCs and the PLA composites prepared therefrom were characterized, providing new information about their mechanical, water vapor barrier, thermal, and morphological properties, in addition to their biodegradation potential. Cast films of these hybrid CNC composites displayed a unique improvement in mechanical properties consistent with recent studies of hybrid CNC mixtures. The maximum strength and the elastic modulus of these hybrid cast films surpassed both nanocomposite materials which incorporated only one CNC type and the unmodified PLA matrix. The extruded films did not show this hybrid effect; however, this may be due to aggregation of the CNCs in these samples. Further study is warranted to determine whether this hybrid effect observed in cast films can be translated into extruded films. Little difference in nanocomposite performance was observed between extruded samples reinforced with wood or tunicate CNCs. Notably, the water vapor permeability of the composites with 1% CNCs was reduced by approximately 60% compared to neat PLA. The primary challenge encountered when combining PLA with the modified CNCs produced in this work was the aggregation of CNCs during the melt-mixing process, particularly as the loading of the CNCs increased from 1 wt.% to 2 wt.%. However, the extruded 1 wt.% composites displayed comparable mechanical and thermal properties to the PLA, while also displaying superior vapor transmission and biodegradation properties, indicating the potential of these materials in sustainable packaging applications.

## Figures and Tables

**Table 1:** Measured length, width, and aspect ratio of the ODA–TA-CNCs and their precursors.

	W-CNC	TA-W-CNC	ODA–TA-W-CNC	T-CNC	TA-T-CNC	ODA–TA-T-CNC
**Length (nm)**	211.8	242.1	260.8	1213.6	1215.0	1238.7
**SD**	±49.1	±60.4	±54.0	±505.1	±454.1	±340.9
**Width (nm)**	20.3	22.1	36.7	20.6	26.9	32.1
**SD**	±6.6	±7.2	±8.4	±6.1	±9.3	±7.4
**Aspect Ratio**	12.5	12.8	7.0	58.9	41.9	38.5
**SD**	±7.9	±7.3	±6.3	±28.8	±25.7	±26.9

All values were obtained from TEM micrographs.

**Table 2:** Designated sample codes used herein.

S-T-PLA	Solution-cast T-CNC 1 wt.% in PLA
S-W-PLA	Solution-cast W-CNC 1 wt.% in PLA
S-H-PLA	Solution-cast H-CNC 1 wt..% in PLA
S-PLA	Solution-cast PLA
E-UP-PLA	Extruded film without k-mixing
E-P-PLA	K-mixed then extruded film
E-1W-PLA	K-mixed then extruded film
E-2W-PLA	K-mixed then extruded film
E-1T-PLA	K-mixed then extruded film
E-2T-PLA	K-mixed then extruded film
E-1H-Pre-PLA	K-mixed W-CNCs and T-CNCs together to form a H-CNC mixture in the PLA matrix, then extruded film
E-2H-Pre-PLA	K-mixed W-CNCs and T-CNCs together to form a H-CNC mixture in the PLA matrix, then extruded film
E-1H-Post-PLA	K-mixed W-CNCs and T-CNCs in separate PLA matrices, then mixed while extruding H-CNC films
E-2H-Post-PLA	K-mixed W-CNCs and T-CNCs in separate PLA matrices, then mixed while extruding H-CNC films

**Table 3:** Thermal properties of the designated composites obtained from TGA.

Composite	Onset Temperature (°C)	Inflection Point (°C)
E-2T-PLA	295	354
E-1T-PLA	303	368
E-2W-PLA	299	360
E-1W-PLA	301	366
E-UP-PLA	306	367
E-P-PLA	304	357
E-2H-Post-PLA	298	355
E-1H-Post-PLA	299	364
E-2H-Pre-PLA	297	353
E-1H-Pre-PLA	300	363

**Table 4:** Thermal properties of the designated composites obtained from DSC.

Sample *	**Tg (°C)**	**Tc (°C)**	**ΔHc (J/g)**	**Tm (°C)**	**ΔHm (J/g)**
E-UP-PLA	62	Not observed	Not observed	150	0.5
E-P-PLA	61	116	8.1	147	8.2
E-2W-PLA	59	121	29.5	148	29.7
E-2T-PLA	60	123	26.2	150	26.5
E-2H-Pre-PLA	59	122	27.3	149	27.6
E-2H-Post-PLA	60	122	26	149	26.5
E-1W-PLA	61	121	25.4	149	27.1
E-1T-PLA	61	121	22	149	23.2
E-1H-Pre-PLA	61	121	22.7	149	24.4
E-1H-Post-PLA	61	120	24.7	148.5	25.2

* Note: These values are consistent for the 2nd–10th heating cycles.

**Table 5:** Measured water vapor transmission properties of all extruded film samples.

Sample	WVTR (g/h/m^2^)	Permeance (g/s/m^2^/Pa) (×10^−7^)	Permeability (g/s/m/Pa) (×10^−11^)
E-UP-PLA	1.1 ± 0.4	2.5 ± 0.1	7.5 ± 2.7
E-P-PLA	1.2 ± 0.1	2.7 ± 0.1	6.8 ± 0.4
E-1T-PLA	0.5 ± 0.1	1.2 ± 0.1	3.1 ± 0.1
E-1W-PLA	0.5 ± 0.1	1.1 ± 0.2	2.9 ± 0.6
E-1H-Pre-PLA	0.6 ± 0.1	1.2 ± 0.1	3.4 ± 0.1
E-1H-Post-PLA	0.8 ± 0.2	1.7 ± 0.5	4.6 ± 1.4
E-2T-PLA	0.7 ± 0.2	1.4 ± 0.4	3.7 ± 1.0
E-2W-PLA	1.2 ± 0.7	2.7 ± 0.2	7.4 ± 4.1
E-2H-Pre-PLA	0.9 ± 0.3	2.0 ± 0.1	5.5 ± 1.7
E-2H-Post-PLA	1.2 ± 0.8	2.7 ± 0.2	7.2 ± 4.4

**Table 6:** Measured mass remaining after anaerobic digestion of the designated samples.

**Sample**	**Mass after Anaerobic Digestion (%)**	**Standard Deviation**
E-UP-PLA	96	±2
E-P-PLA	95	±2
E-1T-PLA	94	±2
E-1W-PLA	92	±3
E-1H-Pre-PLA	92	±3
E-1H-Post-PLA	92	±3
E-2T-PLA	92	±2
E-2W-PLA	90	±2
E-2H-Pre-PLA	91	±4
E-2H-Post-PLA	91	±3

## Data Availability

Not applicable.

## Supplementary Materials

The following are available online at https://www.mdpi.com/article/10.3390/polym13213661/s1, S1: CNC Modification. Figure S1: WCNCs (Left), TA-WCNCs (Center), and ODA-TA-WCNCs (Right). Figure S2-1. Thermal decomposition profiles of designated composites. Figure S2-2. DTGA thermograms of designated extruded samples. Figure S3-1: Measured Toughness and Max Force of solution cast film samples. Figure S3-2: Measured Toughness and Max Force of extruded film samples. Figure S4-1. Measured permeance of all extruded film samples. Figure S4-2: Measured permeability of all extruded film samples. Figure S5. Photographs (iPhone 7) of E-P-PLA (Left), E-1T-PLA (Center), and E-2T-PLA (Right). Table S1. Measured Contact Angle of designated samples. Table S2-1: Measured values obtained from the DSC thermograms of the 1st thermocycle. Table S2-2: Values obtained from subtracting DSC thermograms (i.e. 2nd cycle – 1st cycle). Table S3. Measured mechanical properties of the designated samples.
