# Supplementary material for: Polylactic Acid Cellulose Nanocomposite Films Comprised of Wood and Tunicate CNCs Modified with Tannic Acid and Octadecylamine
Source: Polymers (Basel). 2021 Oct 24;13(21):3661. doi: 10.3390/polym13213661 (PMC8588324; doi:10.3390/polym13213661)
Supplement: Supplementary file 1 [file polymers-13-03661-s001.zip › polymers-1416861-supplementary.pdf]

Supplementary Information

For the manuscript entitled: Polylactic acid cellulose nanocomposites comprised of wood and tunicate CNCs modified with tannic acid and octadecylamine

Matthew J. Dunlop [a, b], Ronald Sabo [c], Rabin Bissessur [b] and Bishnu Acharya [d]

a) Faculty of Sustainable Design Engineering, University of Prince Edward Island, Charlottetown, Canada.

b) Department of Chemistry, University of Prince Edward Island, Charlottetown, Canada.

c) USDA Forest Service, Forest Product Laboratory, Madison Wisconsin, USA.

d) Department of Chemical and Biological Engineering, College of Engineering, University of Saskatchewan, Saskatoon, Canada.

S1: CNC Modification

Dispersions of W-CNC and T-CNC (4000 g at 0.58 wt% CNC) were mechanically mixed at room temperature (~20 ˚C) using a Stir-pak Laboratory Mixer. Power was adjusted to account for the slightly different viscosities of the dispersions to maintain a consistent mixing rate. Next, 20 g of (4-(2-hydroxyethyl)-1-piperazineethanesulfonic acid) (HEPES) was added to the mixing solutions and the pH was adjusted to 8 by the addition of NaOH (aq). Once the pH stabilized, 1.16 g of tannic acid powder was added manually, in small portions over a period of 10 minutes. The dispersions noticeably increased in viscosity as the tannic acid was added, prompting the power to be adjusted again to ensure uniform mixing. The dispersions were allowed to stir for 6 hours after tannic acid addition.

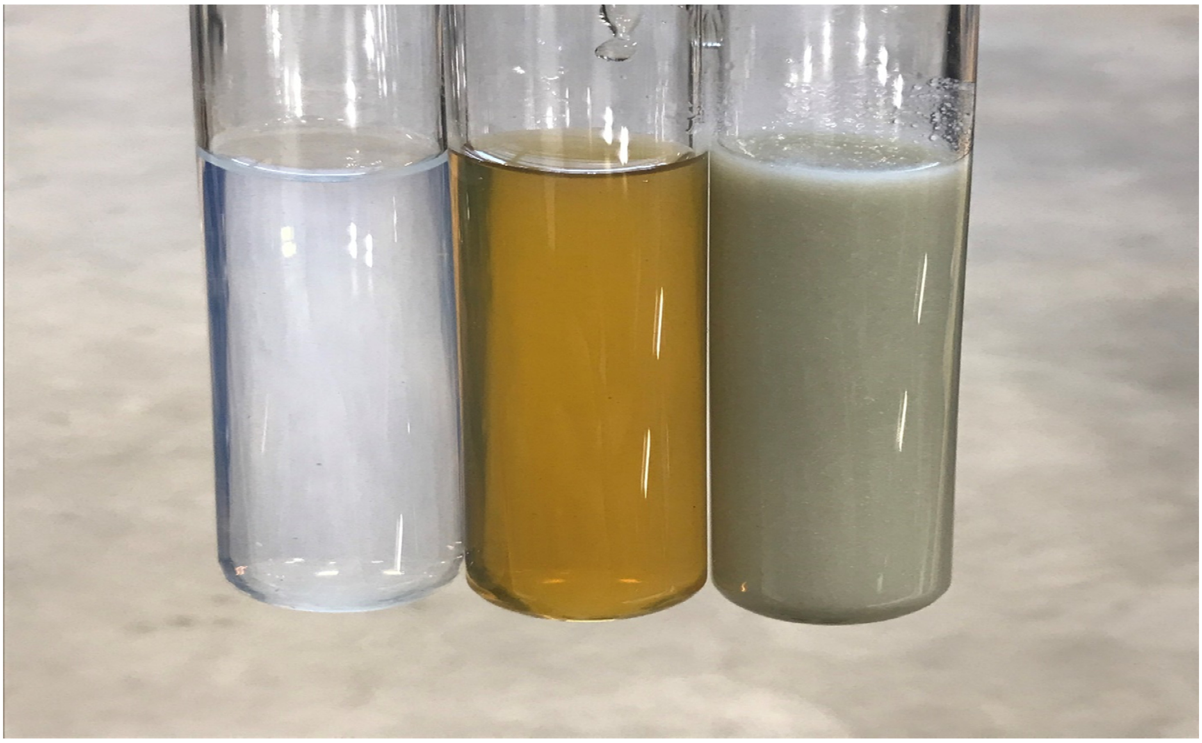

Figure S1: WCNCs (Left), TA-WCNCs (Center), and ODA-TA-WCNCs (Right).

The color became noticeably darker immediately after tannic acid addition as visible in Figure S1. After TA modification, ODA modification was performed by first dissolving 16 g of ODA in 300 mL of hot (70 ˚C) 200 Proof ethanol with stirring. Once homogenous this ODA/ethanol solution was allowed to cool to 40 ˚C and then poured into 2000 g of the TA-CNC dispersions prepared previously, with strong stirring. After the ODA addition, the stirring was continued for 2 hours and additional color change was noted. After 2 hours the stirring was stopped and the dispersions were allowed to settle overnight. A clear phase separation was observed between the now hydrophobic CNCs which collected at the top, and the aqueous solution below. This phase separation was not immediate and took place over a period of hours. By determining the solid content of these dispersions and assuming that all solid content in the dispersion is modified CNC, we were able to use this dispersion as an input for all of the composite preparation described in this work, without any purification or post treatment.

S2: Contact Angle Measurements

Table S1: Measured Contact Angle of designated samples.

Row	Sample	Contact Angle	Comment
1	T-CNC	23.0	Unmodified T-CNC film cast from water
2	W-CNC	18.3	Unmodified W-CNC film cast from water
3	TA-T-CNC	26.7	Tannic acid modified T-CNC film cast from water
4	TA-W-CNC	22.5	ODA-TA modified W-CNC film cast from water
5	ODA-TA-T-CNC	73.8	ODA-TA modified T-CNC film cast benzene
6	ODA-TA-W-CNC	70.4	Tannic acid modified W-CNC film cast from benzene
7	S-T-PLA	82.8	Cast from benzene, contains T-CNC 1wt% in PLA
8	S-W-PLA	83.0	Cast from benzene, contains W-CNC 1wt% in PLA
9	S-H-PLA	80.9	Cast from benzene, contains 1:1 mixture of W-CNC and T-CNC 1wt% in PLA
10	S-PLA	77.7	Cast from benzene, contains only PLA
11	E-UP-PLA	87.8	Extruded Film from unprocessed PLA (no K-Mixing)
12	E-P-PLA	83.7	K-Mixed PLA with Water then Extruded Film
13	E-1W-PLA	82.3	K-Mixed 1% W-CNC and PLA, then Extruded Film
14	E-2W-PLA	84.9	K-Mixed 2% W-CNC and PLA, then Extruded Film
15	E-1T-PLA	86.0	K-Mixed 1% T-CNC and PLA, then Extruded Film
16	E-2T-PLA	83.9	K-Mixed 2% T-CNC and PLA, then Extruded Film
17	E-1H-Pre-PLA	84.1	K-Mixed W-CNCs and T-CNCs together to form 1:1 1% H-CNC mixture in PLA matrix, then Extruded Film
18	E-2H-Pre-PLA	77.0	K-Mixed W-CNCs and T-CNCs together to form 1:1 2% H-CNC mixture in PLA matrix, then Extruded Film
19	E-1H-Post-PLA	80.3	K-Mixed 1% W-CNC and 1% T-CNC into separate PLA matrices, then mixed 1:1 during Extrusion
20	E-2H-Post-PLA	73.1	K-Mixed 2% W-CNC and 2% T-CNC into separate PLA matrices, then mixed 1:1 during Extrusion

Where all CNCs used in Rows 7-20 are assumed to be modified as described in ‘CNC modification’ and the cast film samples in Rows 1-6 were conditioned at 25 ˚C under vacuum for 24 hours prior to analysis.

S3: TGA and DTGA Thermograms

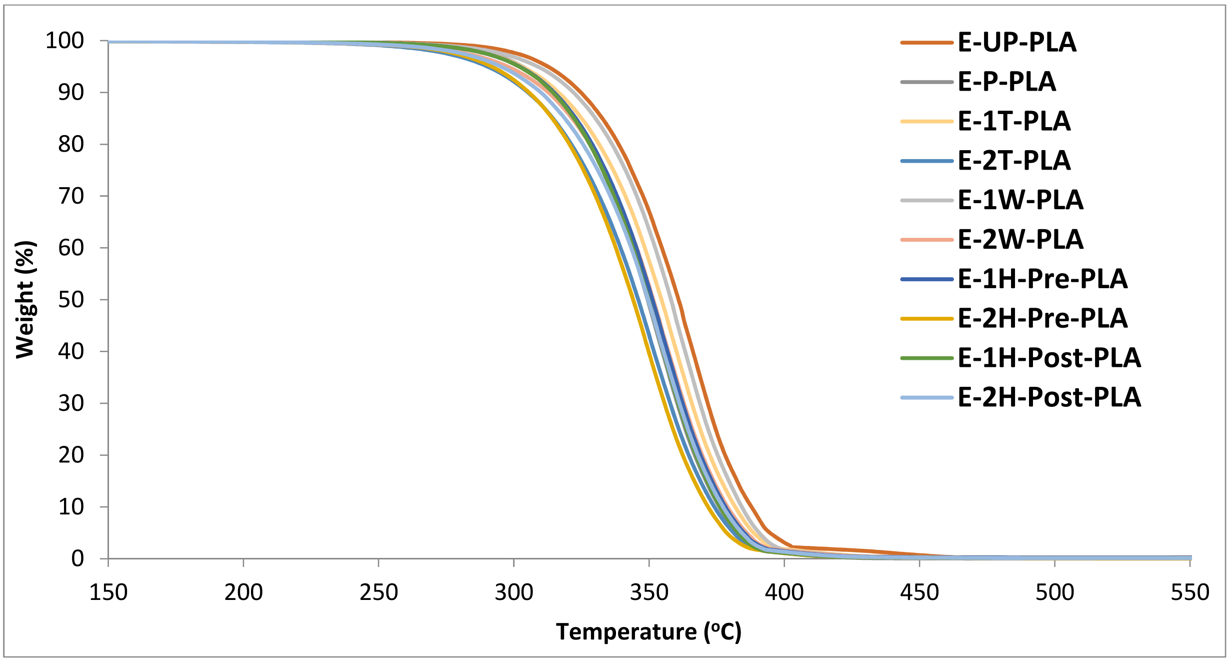

Figure S2.1: Thermal decomposition profiles of designated composites.

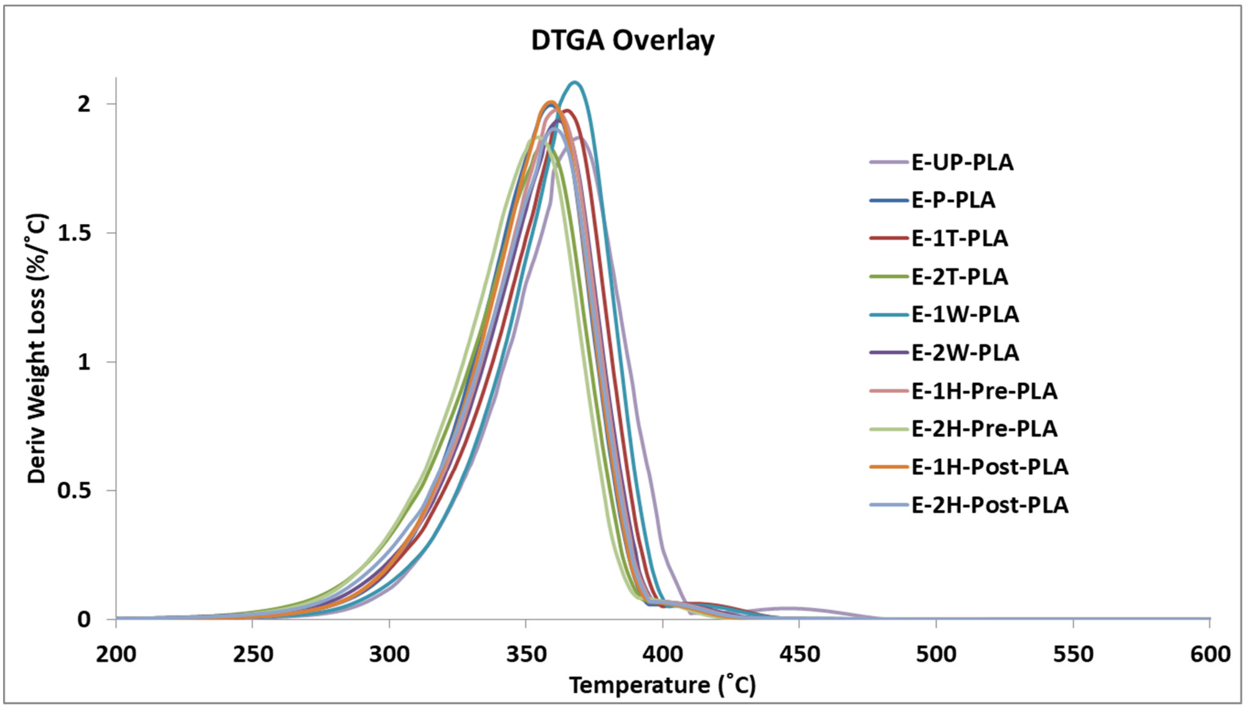

Figure S2.2: DTGA thermograms of designated extruded samples.

S4: DSC Data

Table S2.1: Measured values obtained from the DSC thermograms of the 1st thermocycle.

DSC - 1st Cycle	Tg (°C)	Tc (°C)	ΔHc (J/g)	Tm (°C)	ΔHm (J/g)
E-UP-PLA	66	Not Observed	Not Observed	150	34.3
E-P-PLA	65	Not Observed	Not Observed	149.5	26.6
E-2W-PLA	64	Not Observed	Not Observed	152.1	34.52
E-2T-PLA	65	Not Observed	Not Observed	152.4	31.45
E-2H-Pre-PLA	65	Not Observed	Not Observed	151.9	33
E-2H-Post-PLA	64	Not Observed	Not Observed	152.1	31.1
E-1W-PLA	64	115	19.9	147.7	29
E-1T-PLA	64	115.5	4.3	151.3	28.5
E-1H-Pre-PLA	64	Not Observed	Not Observed	151.6	28.5
E-1H-Post-PLA	64	118	13.2	148	27.8

*Note: These values are obtained from the DSC thermograms of the 1st thermocycle.

Table S2.2: Values obtained from subtracting DSC thermograms (i.e. 2nd cycle – 1st cycle).

Δ DSC (2nd-1st)	Tg (°C)	Tc (°C)	ΔHc (J/g)	Tm (°C)	ΔHm (J/g)
E-UP-PLA	-4	N/A	N/A	0	-33.8
E-P-PLA	-4	N/A	N/A	-2.5	-18.4
E-2W-PLA	-5	N/A	N/A	-4.1	-4.82
E-2T-PLA	-5	N/A	N/A	-2.4	-4.95
E-2H-Pre-PLA	-5.5	N/A	N/A	-2.9	-5.4
E-2H-Post-PLA	-4.5	N/A	N/A	-3.1	-4.6
E-1W-PLA	-3	6	5.5	1.3	-1.9
E-1T-PLA	-3	5.5	17.7	-2.3	-5.3
E-1H-Pre-PLA	-3	N/A	N/A	-2.6	-4.1
E-1H-Post-PLA	-3	2	11.5	0.5	-2.6

*Note: These values are calculated by subtracting the values obtained from the 1st thermocycle from values obtained for the 2nd thermocycle. ** DSC data for the 2nd thermocycle can be found in Figure 4.

S5: Mechanical Data

Table S3: Measured mechanical properties of the designated samples.

Sample Code	Max Force (N)	Modulus (MPa)	Max Strength (MPa)	Toughness (J/m)
S-T-PLA	9.0 ± 1.3	2324.6 ± 193.2	33.8 ± 2.3	0.0033 ± 0.0007
S-W-PLA	19.6 ± 1.4	2004.0 ± 555.7	30.1 ± 2.9	0.0063 ± 0.0018
S-H-PLA	15.5 ± 1.1	2507.8 ± 419.9	38.4 ± 3.6	0.0552 ± 0.0157
S-PLA	16.7 ± 1.0	1731.6 ± 210.9	30.7 ± 3.2	0.1403 ± 0.1098
E-UP-PLA	46.7 ± 4.4	2359.5 ± 549.2	39.7 ± 6.2	0.0989 ± 0.0753
E-P-PLA	39.5 ± 4.7	1721.4 ± 507.8	42.0 ± 5.1	0.0845 ± 0.0419
E-1W-PLA	42.5 ± 3.3	2696.6 ± 411.0	40.0 ± 4.2	0.0231 ± 0.0108
E-2W-PLA	42.7 ± 3.2	2190.1 ± 1050.0	32.8 ± 6.3	0.0081 ± 0.0030
E-1T-PLA	46.4 ± 3.8	2375.8 ± 415.9	36.9 ± 3.4	0.0383 ± 0.0209
E-2T-PLA	40.3 ± 2.8	2042.7 ± 368.1	29.6 ± 4.9	0.0076 ± 0.0048
E-1H-Pre-PLA	41.8 ± 5.1	2371.1 ± 589.7	34.6 ± 4.2	0.0230 ± 0.0160
E-2H-Pre-PLA	41.3 ± 3.4	2395.8 ± 789.9	30.4 ± 4.7	0.0069 ± 0.0058
E-1H-Post-PLA	42.0 ± 2.4	2354.4 ± 685.4	37.6 ± 2.6	0.0308 ± 0.0210
E-2H-Post-PLA	44.7 ± 1.9	2239.9 ± 421.7	28.8 ± 2.7	0.0079 ± 0.0037

*Where 1 and 2 represent the loading of CNC by wt %, all CNCs used are assumed to be modified as described in 'CNC Modification', and all hybrid mixtures are comprised of a 1:1 ratio of W-CNC:T-CNC.

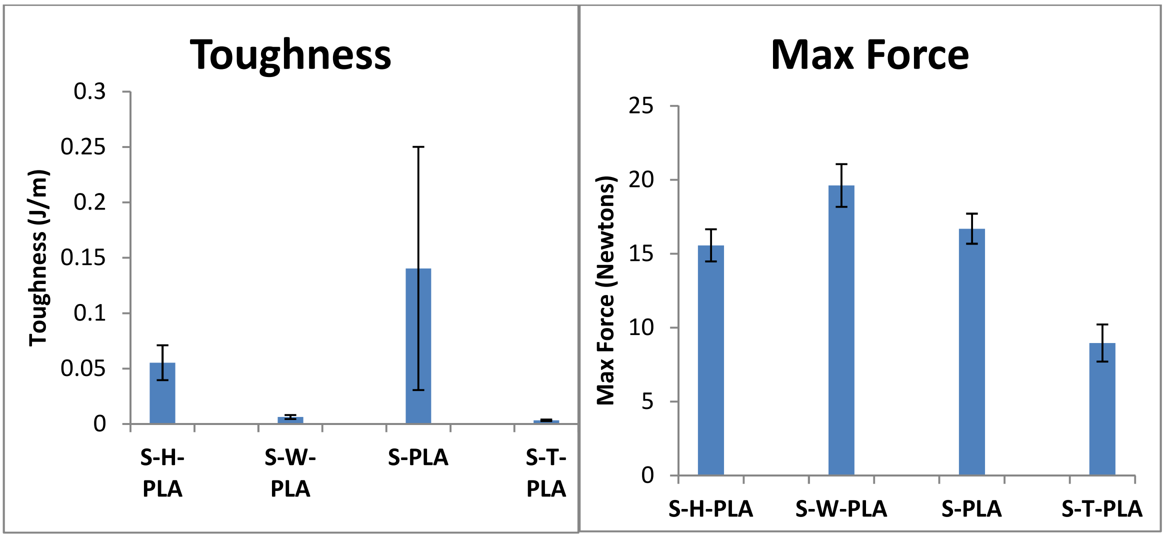

Figure S3.1: Measured Toughness and Max Force of solution cast film samples.

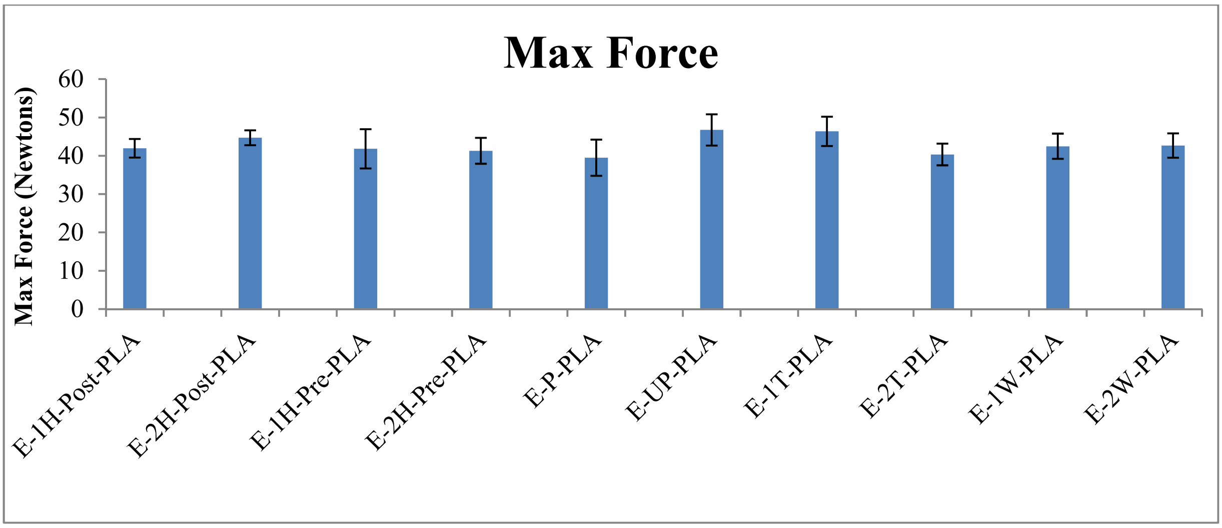

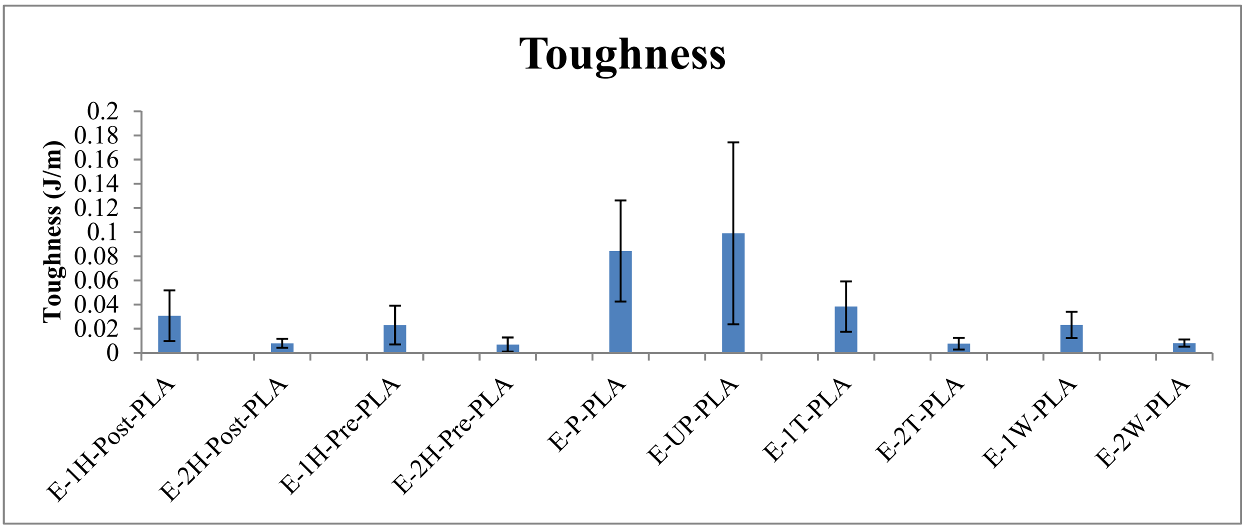

Figure S3.2: Measured Toughness and Max Force of extruded film samples.

S6: Water Vapor Transmission Data

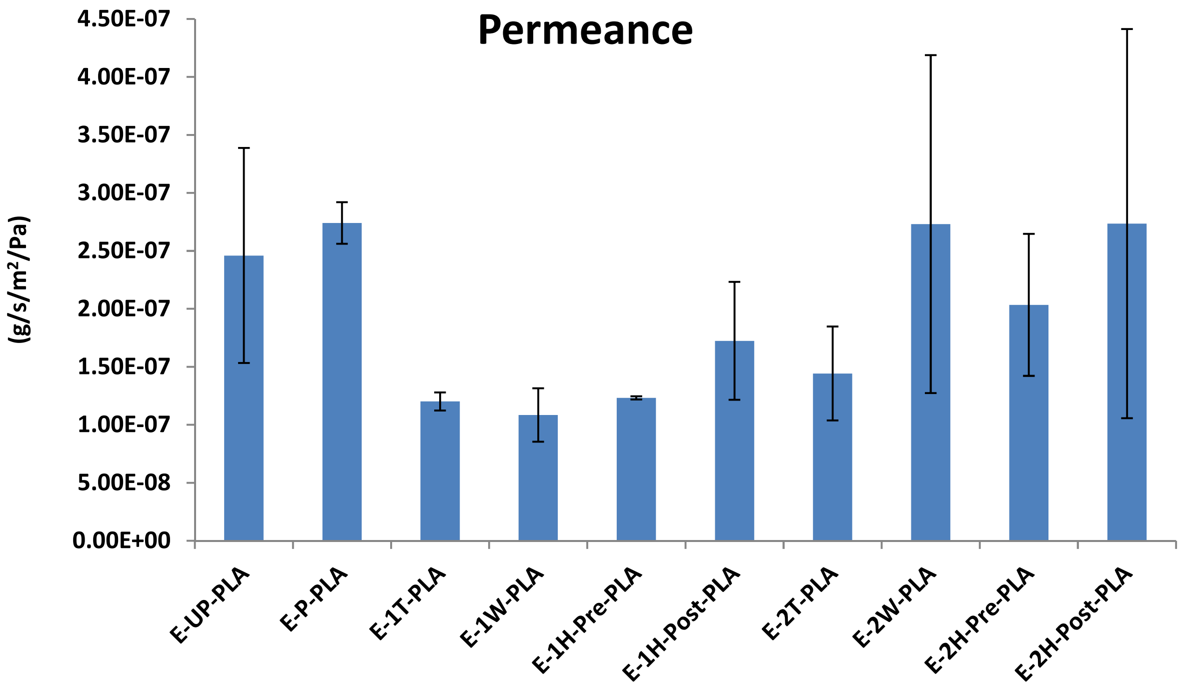

Figure S4.1: Measured permeance of all extruded film samples.

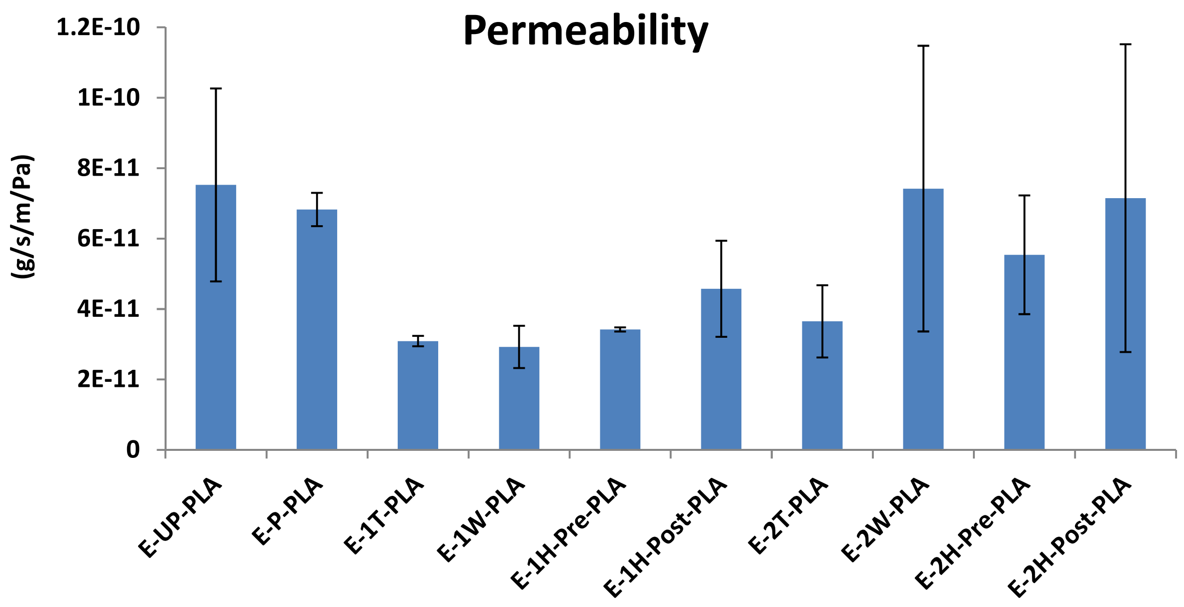

Figure S4.2: Measured permeability of all extruded film samples.

S7: Visual Observations of Extruded Film Samples

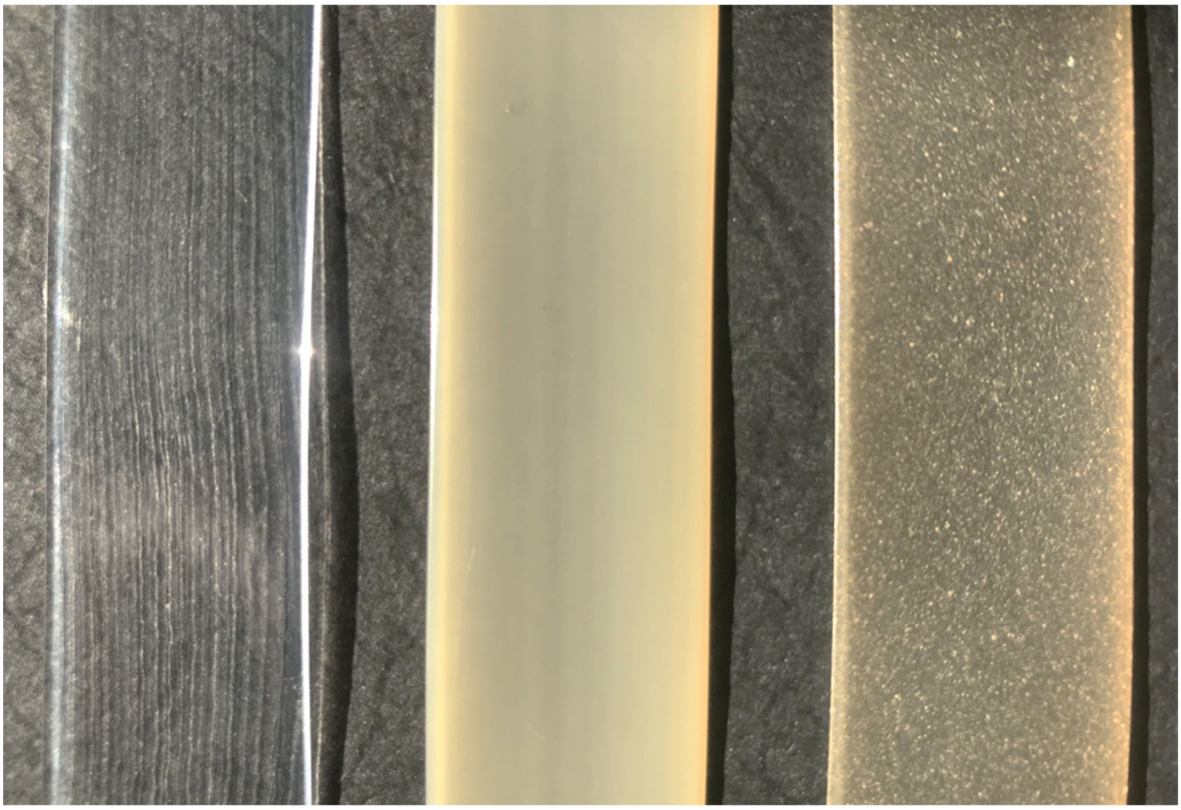

Figure S5: Photographs (iPhone 7) of E-P-PLA (Left), E-1T-PLA (Center), and E-2T-PLA (Right). The samples visible in Figure S7 are representative of all extruded samples prepared in this work. They generally appear homogenous in the case of the PLA samples and samples which incorporate 1 wt% CNC, however samples with 2 wt% CNCs display clear evidence of CNC aggregation with visible aggregates throughout the film samples. Notably, this aggregation appeared more severe in samples containing TCNCs than those which only incorporated WCNCs.
